# Supplementary material for: Exploration of alive-and-ventilator free days as an outcome measure for clinical trials of Resuscitative interventions
Source: PLoS One. 2024 Jul 31;19(7):e0308033. doi: 10.1371/journal.pone.0308033 (PMC11290648; doi:10.1371/journal.pone.0308033)
Supplement: S1 File — (DOCX) [file pone.0308033.s001.docx]

**Exploration of Alive-and-Ventilator Free Days as an Outcome Measure for Clinical Trials of Resuscitative Interventions**

**Data Supplement**

Contents

[**Supplementary Tables** 2](#_Toc167435982)

[Supplementary Table 1: Characteristics of IHCA Observational Cohort 2](#_Toc167435983)

[Additional Details Regarding Simulation Procedures 2](#_Toc167435984)

[**Supplementary Figures:** 5](#_Toc167435985)

[Supplementary Figure 1 (S1): Scenario 2 for AVFD1 5](#_Toc167435986)

[Supplementary Figure 2 (S2): Scenario 2 for AVFD2 6](#_Toc167435987)

[Supplementary Figure 3 (S3): Scenario 3 for AVFD1 7](#_Toc167435988)

[Supplementary Figure 4 (S4): Scenario 3 for AVFD2 8](#_Toc167435989)

[Supplementary Figure 5 (S5): Scenario 4 for AVFD1 9](#_Toc167435990)

[Supplementary Figure 6 (S6): Scenario 4 for AVDF2 10](#_Toc167435991)

[Supplementary Figure 7 (S7): Scenario 5 for AVFD1 11](#_Toc167435992)

[Supplementary Figure 8 (S8) : Scenario 5 for AVFD2 12](#_Toc167435993)

[Supplementary Figure 9 (S9): Simulation Results Based on PART Trial Bootstrapped Samples with AVFD1 13](#_Toc167435994)

[Supplementary Figure 10 (S10): Simulation Results Based on PART Trial Bootstrapped Samples with AVFD2 14](#_Toc167435995)

# **Supplementary Tables**

## Supplementary Table 1: Characteristics of IHCA Observational Cohort

|  | Overall, N = 534 | ROSC Achieved, N = 201 | ROSC Not Achieved, N = 333 |
| --- | --- | --- | --- |
| Age (mean, SD) | 69 ± 15 | 69 ± 15 | 70 ± 16 |
| Biologic Sex |  |  |  |
| Male (n, %) | 296 (55%) | 115 (57%) | 181 (54%) |
| Female (n, %) | 238 (45%) | 86 (43%) | 152 (46%) |
| Race |  |  |  |
| White (n, %) | 102 (19%) | 31 (15%) | 71 (21%) |
| Black (n, %) | 199 (37%) | 76 (38%) | 123 (37%) |
| Other/Unknown (n, %) | 236 (44%) | 95 (47%) | 141 (42%) |
| Ethnicity |  |  |  |
| Hispanic (n, %) | 166 (31%) | 67 (33%) | 99 (30%) |
| Non-Hispanic (n, %) | 312 (58%) | 115 (57%) | 197 (59%) |
| Elixhauser Comorbidity Score (mean, SD) | 4.93 ± 2.79 | 5.32 ± 2.81 | 4.60 ± 2.74 |
| Days Hospitalized prior to event (mean, SD) | 9 ± 12 | 9 ± 11 | 10 ± 12 |
| ICU admission prior to event (n, %) | 206 (39%) | 142 (71%) | 64 (19%) |
| Length of post-event hospital stay (mean, SD) |  | 9 ± 13 |  |
| Hospital mortality (n, %) |  | 159 (79%) |  |

## Additional Details Regarding Simulation Procedures

**Details about Simulation Studies and Analysis**

1. Model based simulations

The model-based simulations were conducted using a two-part statistical model. The first part uses a multinomial regression model to randomly generate a value for *Y_i_*, a three-category variable denoting whether the *i^th^* subject failed to achieve ROSC (*Y_i_ =* 1), died after ROSC (*Y*_i_ = 2) or survived to 28 days (*Y_i_* = 3). Let *p_1i_* = *Pr(Y_i_ = 1)*, *p_2i_ = Pr(**Y_i_ = 2)* and *p_3i_ = Pr(Y_i_ = 3) = 1 - p_1i_ - p_2i_*. Then the multinomial regression model is expressed as:

$$\log\left( \frac{p_{1i}}{p_{3i}} \right)=\theta_{1}+\theta_{2}x_{i}$$

$$\log\left( \frac{p_{2i}}{p_{3i}} \right)=\theta_{2}+\theta_{3}x_{i}$$

where *x_i_* denotes treatment arm for the *i^th^* subject (0 =control, 1 = intervention).

If the *i^th^* subject is a survivor (*Y_i_*=3), the second part of the two-part statistical modeling approach uses a beta-binomial regression model to randomly generate *Z_i_*, the number of ventilation-free days through day 28. The beta-binomial model was used to account for overdispersion in the count data relative to a binomial distribution. We assume that *Z_i_* follows a beta-binomial distribution with parameters (*n*, $\alpha_{i}$, $\beta_{i}$) where *n* = 28 days. Reparametrizing so that $\pi_{i}= \frac{\alpha_{i}}{\alpha_{i}+\beta_{i}}$and denoting the over-dispersion parameter as $\phi_{i}$= $\frac{1}{\alpha_{i}+\beta_{i}}$, the beta-binomial regression model is expressed as:

$$\log\left( \frac{\pi_{i}}{1-\pi_{i}} \right)=\gamma_{1}+\gamma_{2}x_{i}$$

$$\log(\phi_{i})=\delta_{1}+\delta_{2}x_{i}.$$

For the simulation studies, the parameter values for the two parts: ${(\theta}_{1},\theta_{2}$ $\theta_{3}$, $\theta_{4})$ and ($\gamma_{1},\gamma_{2},\delta_{1},\delta_{2}),$ were set as specified below to satisfy the assumptions in each scenario in Table 1 of the paper.

**Supplemental Table 2**: Simulation parameter settings under each scenario for the two-part statistical model used to simulate the data

| **Scenario** | **Multinomial regression parameters** | | | | **Beta-Binomial regression parameters** | | | |
| --- | --- | --- | --- | --- | --- | --- | --- | --- |
|  | $\theta_{1}$ | $\theta_{2}$ | $\theta_{3}$ | $\theta_{4}$ | $\gamma_{1}$ | $\gamma_{2}$ | $\delta_{1}$ | $\delta_{2}$ |
| 1 | 2.05 | -0.66 | 1.32 | -0.33 | 0.75 | 0.35 | 0.28 | 0.42 |
| 2 | 2.05 | -0.18 | 1.32 | 0.29 | 0.75 | 0 | 0.28 | 0 |
| 3 | 2.05 | -0.18 | 1.32 | 0.29 | 0.75 | -0.31 | 0.28 | -0.21 |
| 4 | 2.05 | -0.31 | 1.32 | -0.13 | 0.75 | 0.17 | 0.28 | 0.17 |
| 5 | 2.05 | -0.40 | 1.32 | 0.01 | 0.75 | 0.17 | 0.28 | 0.17 |

The simulations were conducted as follows:

1. Generate a random value for *Y_i_* given *x_i_* from the multinomial regression model and denote this as *Y_i_**. If *Y_i_** = 1, then *AVFD2_i_* = -1 (failed to achieve ROSC); if *Y_i_**=2, then *AVFD2_i_* = 0 (death after ROSC); if *Y_i_**=3 (survived), then draw a random *Z_i_* from the beta-binomial distribution, *Z_i_** and *AVFD2_i_* = *Z_i_**, where *AVFD2_i_* is the simulated AVFD2 value for the *i^th^* subject.
2. For *AVFD1_i_*, the simulated AVFD1 value for the *i^th^* subject, set *AVFD1_i_* = 0 if *AVFD2_i_* = -1; otherwise *AVFD1_i_* = *AFVD2_i_* .
3. Repeat steps 1-2 until the desired total sample size is achieved.
4. Analyze the data using the different statistical methods and record the p-value for the intervention effect.
5. Repeat steps (1) – (4) for 1000 times.
6. Compute power or type I error rate as the proportion of simulated data sets where the two-sided p-value for the intervention effect is < 0.05.

2. Bootstrap simulations:

1. Sample with replacement from the PART trial data until the desired sample size has been reached.
2. Analyze the data using the different statistical methods and record the p-value for the intervention effect.
3. Repeat steps (1) – (2) for 1000 times.
4. Compute power or type I error rate as the proportion of data sets where the two-sided p-value for the intervention effect is < 0.05.

Simulation analysis software:

All simulations were performed in R version 4.2.2. The T-test was performed with the R function *t.test*, Wilcoxon test with *wilcox.test* function, proportional odds regression model with *polr* function from the R package MASS, and the two-part model with R functions *glm*, *multinom*, and *betabin* functions from the R packages stats, nnet, aod respectively.

# **Supplementary Figures:**

## Supplementary Figure 1 (S1): Scenario 2 for AVFD1

## Supplementary Figure 2 (S2): Scenario 2 for AVFD2

## Supplementary Figure 3 (S3): Scenario 3 for AVFD1

## Supplementary Figure 4 (S4): Scenario 3 for AVFD2

## Supplementary Figure 5 (S5): Scenario 4 for AVFD1

## Supplementary Figure 6 (S6): Scenario 4 for AVDF2

## Supplementary Figure 7 (S7): Scenario 5 for AVFD1

## Supplementary Figure 8 (S8) : Scenario 5 for AVFD2

## Supplementary Figure 9 (S9): Simulation Results Based on PART Trial Bootstrapped Samples with AVFD1

## Supplementary Figure 10 (S10): Simulation Results Based on PART Trial Bootstrapped Samples with AVFD2
